# Antibacterial and Antibiofilm Activity of TheraBreath™ Oral Rinses: An In Vitro Study

**DOI:** 10.3390/dj13090383

**Published:** 2025-08-24

**Authors:** Zaid A. Aboona, Laura A. Young, Joshua J. Thomson

**Affiliations:** 1 Department of Biology, University of Detroit Mercy, Detroit, MI 48221-3038, USA; 2 Division of Integrated Biomedical Sciences, School of Dentistry, University of Detroit Mercy, Detroit, MI 48208-2576, USA

**Keywords:** biofilms, antibacterial agents, *Streptococcus mutans*, saliva, mouthwashes, anti-infective agents, local

## Abstract

**Background/Objectives:** There are many commercial mouthrinses, used for a variety of purposes, including antiseptic activity. The objective of this study was to determine the antibacterial activity of various TheraBreath™ oral rinses against the cariogenic bacterium, *Streptococcus mutans*, and saliva-derived microbial communities, and their antibiofilm activity against *S. mutans* in vitro biofilms. **Methods:** Bactericidal activity against planktonic *S. mutans* was assessed by colony counting after 30 and 2 min exposures to mouthrinses. Ten saliva samples were exposed to mouthrinses for 30 s and plated aerobically on blood agar and Mitis Salivarius agar. Mature biofilms of *S. mutans* were treated with mouthrinses for 15 min followed by fluorescent vitality staining and polysaccharide measurement, followed by crystal violet staining for measurement of total biofilm remaining. Statistical analysis was performed using Kruskal–Wallis with Dunn’s multiple comparisons test comparing all mean ranks (α = 0.05). **Results:** TheraBreath™ Fresh Breath, Healthy Smile, and Dry Mouth exhibited no significant antibacterial activity. TheraBreath™ Healthy Gums showed antibacterial activity against *S. mutans* and microbes from saliva samples similar to Listerine^®^ Naturals at all exposure times. Whitening Fresh Breath showed intermediate killing of *S. mutans* after 30 min in liquid but not after 2 min or against salivary microbes. Live/Dead fluorescence vitality staining showed that Healthy Gums and Whitening Fresh Breath had antibacterial activity against mature biofilms of *S. mutans* statistically similar to Listerine^®^ Naturals and Colgate^®^ Total; however, Whitening Fresh Breath did not have significant killing compared to PBS. **Conclusions:** TheraBreath™ Healthy Gums demonstrated similar antiseptic activity levels to other antiseptic-claiming commercial rinses. Whitening Fresh Breath was comparable but unable to kill in short exposure times.

## 1. Introduction

Dental diseases like caries and periodontal conditions such as gingivitis and periodontitis are some of the most common chronic health conditions worldwide [1]. Dental caries, more commonly known as tooth decay, is a highly prevalent disease that affects almost everyone at some point. It is the most common chronic disease of childhood [2] and is signified by the breakdown of enamel on the surface of teeth as the result of acid production by bacteria. Periodontal (gum) diseases are inflammatory conditions that begin with microbial accumulation on tooth surfaces that begin to invade the tissues that hold teeth in place. Gingivitis is an early, reversible form of periodontal disease characterized by red, swollen gums, while periodontitis is a chronic condition that leads to irreversible destruction of periodontal ligament fibers and supporting bone loss. Nearly half of adults in the United States over the age of 30 have some degree of periodontitis, showing that it is not only a dental health problem but also a public health concern [3]. Alongside dental caries, these diseases are the leading causes of tooth loss and can significantly impact systemic health if left untreated.

Prevention of dental caries and periodontal disease is primarily achieved through physical removal of bacteria from tooth surfaces through routine oral hygiene practices such as brushing, flossing, and professional dental prophylactic cleaning [4]. Usage of mouthrinses can provide additional preventative benefit by killing oral microbes, reducing dental plaque biofilm, or providing fluoride, which in turn reduces the likelihood of developing periodontal disease or caries [5,6]. While mouthrinses are not designed to be an alternative to brushing and flossing, they can be used as an additional line of defense to protect the oral cavity, being particularly effective in areas that may be missed by mechanical removal techniques [7,8].

Mouthrinses vary in composition and intended usage or benefit, which can usually be determined based on their active ingredients. Over-the-counter mouthrinses may contain antiseptic agents, natural compounds, essential oils, and/or fluoride. Oral rinses are also available and aim to help control dry mouth or oral sores [9]. Studies have shown that mouthrinses can be effective in the control of bacteria, plaque, and dental disease [5,6,9,10]. In addition to controlling bacteria, several oral rinses have shown clinical benefit in freshening breath, reducing gingivitis, and reducing the risk of cavities [11]. For example, mouthrinses that contain chlorhexidine as an active ingredient used consistently over a four-to-six-week period can show significant reduction in both plaque and gingivitis [9].

A mouthrinse that is advertised as antiseptic should be able to kill oral microorganisms. An additional desirable quality of an antiseptic mouthrinse is the ability to kill bacteria protected in a biofilm, since dental diseases generally arise from biofilm-associated bacteria [12]. *Streptococcus mutans*, a cariogenic bacterial pathogen that is highly associated with initiation of dental caries [13], provides a model target for determining the antibacterial and antibiofilm activity of mouthrinses. *S. mutans* is a key contributor to the caries initiation process due to its ability to produce acid in the presence of dietary sugars and form biofilms on the surface of teeth (dental plaque). *S. mutans* can metabolize carbohydrates, resulting in acid production that leads to the demineralization of tooth enamel at a pH lower than 5.5 [14]. *S. mutans* can also produce soluble and insoluble extracellular polysaccharides (EPS), which make up the protective matrix of the biofilm and also allow for other bacteria to join the biofilm, resulting in the development of multispecies biofilms on the surface of the tooth [15,16].

Commercial mouthrinses are marketed for various uses in oral hygiene and some have formulations that have not been assessed for antibacterial and antibiofilm activity. One such brand is TheraBreath™, which has marketed mouthrinses for various applications, yet no studies have investigated their antibacterial and antibiofilm efficacy. Therefore, in this study, we aimed to determine the in vitro bactericidal and antibiofilm activity of five TheraBreath™ mouthrinses. Bactericidal activity was assessed by exposing planktonic *S. mutans* and saliva-derived microbial communities to the various oral rinses, while the antibiofilm activity of TheraBreath™ mouthrinses was assessed using monospecies *S. mutans* in vitro biofilms as a model of cariogenic plaque biofilm.

## 2. Materials and Methods

### 2.1. Liquid Killing Assay for Antibacterial Activity

Streptomycin-resistant *Streptococcus mutans* (ATCC^®^ 25175) was grown in BHI broth (Becton Dickinson and Company, Sparks, MD, USA) overnight at 37 °C/5% CO_2_. Optical density at 600 nm (OD_600_) was measured and cultures were centrifuged and adjusted to an OD_600_ = 0.5 in 1X phosphate-buffered saline (PBS) (~5.0 × 10^8^ CFU/mL). This culture was diluted 1:10 in PBS and 50 µL was added to 200 µL of PBS or mouthrinse (final concentration of 80% mouthrinse). Mixtures were incubated with rotation at 37 °C for 30 min or 2 min. *S. mutans* mixtures were plated on BHI agar (Becton Dickinson and Company, Sparks, MD, USA) containing 500 µg/mL streptomycin and grown for 48 h at 37 °C/5% CO_2_. Surviving bacteria were enumerated by colony counting. Percent survival was calculated by the number of surviving colonies in the treatment condition/PBS control × 100. Strains were tested a minimum of 3 times in separate experiments. Mouthrinses used in this study, along with their ingredients, are listed in Table 1.

### 2.2. Collection and Treatment of Human Saliva Samples

This study was reviewed and approved by the University of Detroit Mercy Institutional Review Board (IRB No. 24-25-42). Following informed consent, ten volunteers were provided with orthodontic wax and instructed to chew on it for a period of five minutes to stimulate salivation and were asked to provide the sample in sterile collection cups. Each collected sample was immediately divided for treatment with PBS, TheraBreath™ Whitening Fresh Breath, TheraBreath™ Healthy Gums, or Listerine^®^ Naturals. Then, 100 µL of stimulated saliva was added to a tube containing 900 µL of mouthrinse or PBS (90% final mouthrinse concentration). The sample was mixed by inversion for 30 s followed by immediate dilution in PBS. Treatments with the remaining mouthrinses were completed one by one. Post treatment, samples were plated onto BHI agar supplemented with 5% sheep blood (HemoStat Laboratories, Dixon, CA, USA), 0.5 µg/mL vitamin K1 (Sigma Aldrich, St. Louis, MO, USA), and 5 µg/mL hemin (Thermo Scientific, Fair Lawn, NJ, USA) and Mitis Salivarius agar (Becton Dickinson and Company, Sparks, MD, USA) supplemented with 1% potassium tellurite (Sigma Aldrich, St. Louis, MO, USA) for oral streptococci. These plates were subsequently incubated at 37 °C/5% CO_2_. Surviving bacteria were enumerated by colony counting. Percent survival was calculated by the number of surviving colonies in the treatment condition/PBS control × 100.

### 2.3. Biofilm Setup and Treatment

Overnight broth cultures of streptomycin-resistant *Streptococcus mutans* ATCC^®^ 25175 (American Type Culture Collection, Manassas, VA, USA) were prepared in BHI broth with 500 µg/mL streptomycin from colonies and incubated for 16 h at 37 °C/5% CO_2_. Overnight cultures were adjusted to an OD_600_ of 0.1. Then, tissue culture-treated 24-well plates containing Brain Heart Infusion (BHI) broth, 1% sucrose, 500 µg/mL streptomycin, and 1 µM Cascade Blue-conjugated 10,000 MW dextran (Life Technologies Corporation, Eugene, OR, USA) were inoculated with *S. mutans* to a final OD_600_ = 0.01 and grown for 24 h at 37 °C/5% CO_2_. After 24 h of growth, biofilm supernatants containing non-adherent and loosely adherent bacteria were removed using a Vacusip (NTEGRA Biosciences Corp., Hudson, NH, USA). Wells were washed twice with 1 mL of PBS by swirling gently. Following, 500 µL of mouthrinses was added to each biofilm well and shaken at 150 rpm for 15 min on an orbital shaker. Then, treatments were removed by Vacusip and washed 2 times with 1 mL of PBS. Biofilm killing was analyzed via fluorescent Live/Dead staining using SYTO9 (Life Technologies Corporation, Eugene, OR, USA) and propidium iodide (Sigma Aldrich, St. Louis, MO, USA). Extracellular polysaccharide (EPS) content of the biofilm was measured via Cascade Blue-conjugated Dextran. Cascade blue-labeled dextran was added during biofilm growth to assess the extracellular polysaccharide content of the biofilms after treatment. The fluorescently labeled dextran acts as an acceptor for glucosidic bonds, becoming incorporated into the biofilm matrix during biofilm development.

### 2.4. Live/Dead Fluorescent Vitality Staining

**Figure A1:**
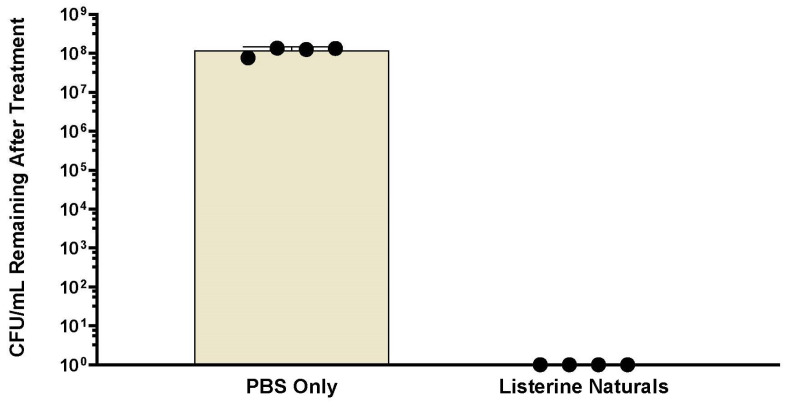
Colony-forming units (CFU) remaining after 4-h treatment of *S. mutans* biofilms. Dots represent individual biofilms. Dots on the x-axis represent zero retrieved CFU from biofilm after treatment.

**Figure A2:**
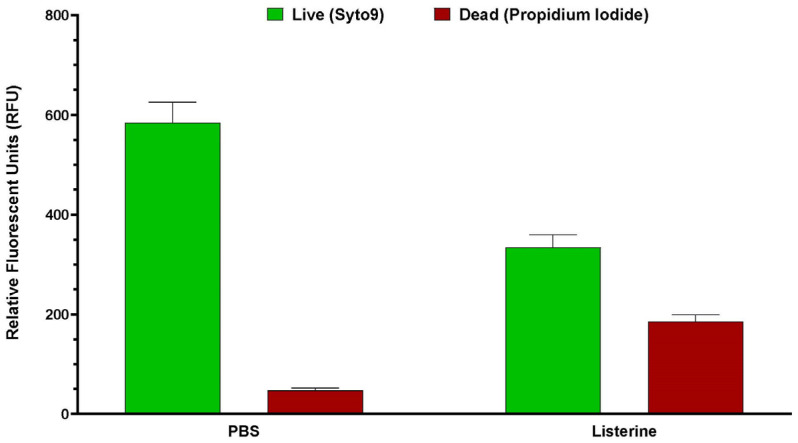
Live and dead relative fluorescent intensity of *S. mutans* biofilms after 4 h treatment.

**Figure A3:**
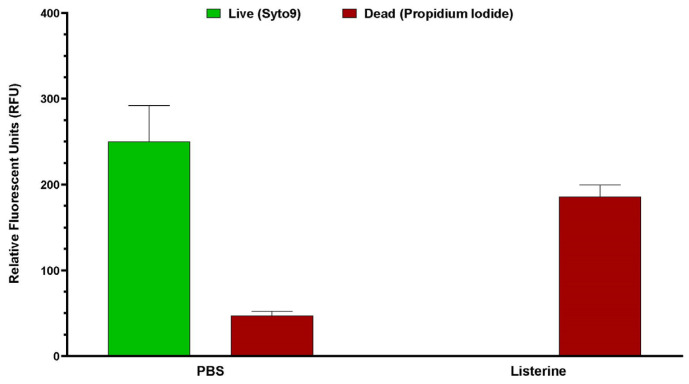
Live and dead relative fluorescent intensity of *S. mutans* biofilms after 4 h treatment with adjustment to subtract background SYTO9 fluorescence.

Post-treatment biofilm vitality was assessed using 250 µL of Live/Dead stain consisting of 2 µM SYTO9 and 20 µM propidium iodide. Biofilms were incubated in the stain for 15 min in the dark. Fluorescence was measured using a Spark microplate reader (TECAN, Grodig, Austria) at five spots within the biofilm: SYTO9 (ex. 485 nm, em. 530 nm), propidium iodide (ex. 485 nm, em. 630 nm), and Dextran (ex. 400 nm, em. 420 nm). The average fluorescent intensity of the five readings was used for each well of the biofilm plate. Experiments were conducted at least three times. Previous experiments have shown that Listerine-treated biofilms produce no surviving bacteria on colony count (Appendix A, Figure A1, Figure A2 and Figure A3). Therefore, for each experiment, the background SYTO9 fluorescent intensity measured in the Listerine^®^-treated biofilms was subtracted from the SYTO9 intensity of all other treatments. If this led to a number less than zero, it was set to zero. Live (SYTO9) and Dextran fluorescent intensities from each experiment were normalized to fluorescent intensities from the PBS-treated biofilms, while the dead fluorescent intensities in each experiment were normalized as a percentage of the respective intensities of the Listerine^®^-treated biofilms. Live/Dead ratios were calculated for each treated biofilm for each experiment by dividing the average live fluorescent intensity for each well by the average dead fluorescent intensity of the well. Live/Dead ratios are displayed as a percentage of the Live/Dead ratio of PBS-treated biofilms.

### 2.5. Crystal Violet Staining

After fluorescent analysis, the stain was removed, and biofilms were heat-fixed at 80 °C for 30 min prior to crystal violet staining for determination of total biofilm remaining after treatment. Briefly, 1 mL of 0.5% crystal violet was added to each well and incubated for 15 min at room temperature. Crystal violet was then removed, and wells were rinsed gently with water until rinses were clear of excess stain. To solubilize biofilm-associated crystal violet, 1 mL of 33% acetic acid was added and incubated for 15 min at room temperature with shaking at 100 rpm. The solubilized stain was transferred to a 96-well plate, serial 1:2 dilutions were made using water, and absorbance at 570 nm was measured using a microplate reader. Absorbance readings were normalized as a percentage of the PBS-treated biofilm for each experiment.

### 2.6. Statistical Analysis

Analysis was completed using GraphPad Prism version 10.4.0 for Windows (GraphPad Software, Boston, MA, USA, www.graphpad.com). Datasets were analyzed to test the assumption of normal distribution. Significance was determined using Kruskal–Wallis followed by Dunn’s multiple comparisons test comparing all mean ranks. A *p* value lower than 0.05 was considered statistically significant.

## 3. Results

### 3.1. Bactericidal Activity Against Streptococcus mutans

**Figure 1:**
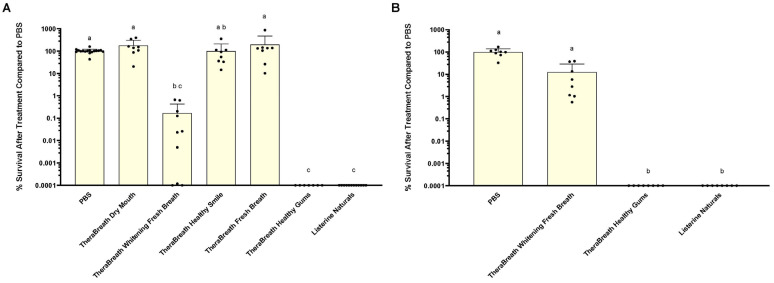
Antimicrobial activity of TheraBreath™ mouthrinses against planktonic *S. mutans*. Percent (%) survival of planktonic *S. mutans* after exposure to mouthrinses for (**A**) 30 min or (**B**) 2 min. Percent survival was calculated by number of surviving colonies in the treatment condition/PBS control × 100. Strains were tested a minimum of 3 times in separate experiments. Individual dots on columns represent a single experimental sample. Dots on the *x*-axis represent recovery of colonies from samples below the limit of detection. Statistical analysis was performed using Kruskal–Wallis test with Dunn’s multiple comparisons test comparing all mean ranks. Lowercase letters denote statistically similar treatments.

To assess the antibacterial activity of TheraBreath™ mouthrinses, bactericidal activity was measured against the cariogenic pathogen, *S. mutans*, in liquid. Approximately 10^7^ CFU/mL *S. mutans* were exposed to PBS, Listerine^®^ Naturals, or TheraBreath™ mouthrinses. Percent survival compared to remaining CFU/mL after treatment in PBS is shown in Figure 1. All TheraBreath™ mouthrinses were tested for antibacterial activity after 30 min exposure (Figure 1A). Statistical analysis was performed using Kruskal–Wallis followed by Dunn’s multiple comparisons test. Following post-test analysis, there was no statistical difference in percent survival after 30 min treatment between PBS and three TheraBreath™ mouthrinses: Dry Breath, Healthy Smile, and Fresh Breath. TheraBreath™ Healthy Gums and Listerine^®^ Naturals both exhibited bactericidal activity resulting in zero recoverable colonies at the lowest tested dilution (>99.9999% killing). TheraBreath™ Whitening Fresh Breath showed more inconsistent bactericidal activity after 30 min, yet was statistically similar to TheraBreath™ Healthy Gums and Listerine^®^, as well as TheraBreath™ Healthy Smile, which exhibited no bactericidal activity.

The 30 min exposure results identified TheraBreath™ mouthrinses that have bactericidal activity, but TheraBreath™ mouthrinse instructions indicate usage for shorter time periods of 0.5 (Healthy Gums) to 2 min (Whitening Fresh Breath), depending on formulation. Therefore, we tested candidate antibacterial TheraBreath™ mouthrinses for bactericidal activity after 2 min exposure to liquid *S. mutans* (Figure 1B). TheraBreath™ Healthy Gums and Listerine^®^ Naturals both completely killed all *S. mutans* to below the limit of detection (>99.9999% killing), which was significant compared to PBS (*p* < 0.0001) and TheraBreath™ Whitening Fresh Breath (*p* = 0.0315). TheraBreath™ Whitening Fresh Breath treatment exhibited low levels of bactericidal activity in some experiments but was not significantly different from the PBS control treatment.

### 3.2. Bactericidal Activity Against Saliva-Derived Microbial Communities

Tests to determine bactericidal activity against planktonic *S. mutans* highlighted the potential of TheraBreath™ Healthy Gums, and to a lesser extent, TheraBreath™ Whitening Fresh Breath. We next aimed to determine the broader bactericidal activity of these mouthrinses against microbes from stimulated saliva samples. Immediately after collection, each saliva sample was mixed and separated for 30 s treatment with PBS, Listerine^®^ Naturals, TheraBreath™ Whitening Fresh Breath, and TheraBreath™ Healthy Gums. A 30 s exposure time was chosen to reflect the instructions of TheraBreath™ Healthy Gums.

**Figure 2:**
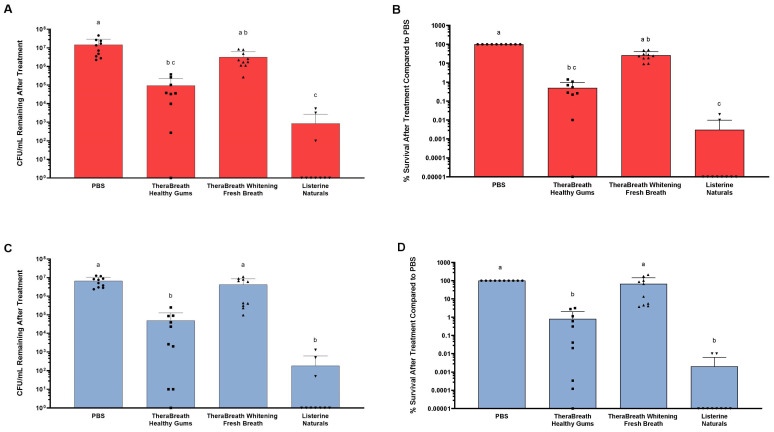
Antimicrobial activity of TheraBreath™ mouthrinses against oral microbes from saliva samples. Saliva-derived microbes were exposed to mouthrinses for 30 s and percent survival after treatment was assessed by colony count on two types of media, (**A**,**B**) blood agar and (**C**,**D**) Mitis Salivarius agar. Results are displayed as (**A**,**C**) colony-forming units per milliliter (CFU/mL) remaining after treatment and (**B**,**D**) percent (%) survival compared to PBS treatment. Percent survival was calculated by the number of surviving colonies in treatment groups divided by PBS (control group) × 100 for each individual saliva sample. Individual dots on columns represent a single experimental sample. Dots on the *x*-axis represent recovery of colonies from samples below the limit of detection. Statistical analysis was performed using Kruskal–Wallis test with Dunn’s multiple comparisons test comparing all mean ranks. Lowercase letters denote statistically similar treatments.

Growth media used after the bactericidal assays were blood agar (Figure 2A,B) and MS agar (Figure 2C,D). Blood agar supports the growth of a broad range of salivary microbes, while MS agar is more selective for *Streptococcus* species like *S. mutans*. On both growth media, Listerine^®^ Naturals had the strongest antibacterial effect, eliminating nearly all bacteria in each sample. TheraBreath™ Healthy Gums was statistically similar to Listerine^®^ Naturals for killing microbes that grew on blood and MS agar (*p* = 0.8041) but it was not able to consistently kill all bacteria to the same level as Listerine^®^ Naturals. TheraBreath™ Healthy Gums had statistically higher levels of bactericidal activity against oral streptococci compared to TheraBreath™ Whitening Fresh Breath (*p* = 0.0418), but percent survival was statistically similar between the two mouthrinses for microbes that grew on blood agar (*p* = 0.2719). TheraBreath™ Whitening Fresh Breath displayed little bactericidal activity and was not significantly different to the PBS control for percent survival of microbes on both agars.

### 3.3. Antibiofilm Activity Against Mature Streptococcus mutans Biofilms

While TheraBreath™ Healthy Gums and Whitening Fresh Breath were the only two TheraBreath™ mouthrinses that showed any detectable level of antibacterial activity, it may not be present against bacteria protected within surface-associated biofilms. Additionally, other formulations, even though they are not bactericidal, may have antibiofilm effects, such as removal of biofilm structure or extracellular matrix. To test the antibiofilm effects of the various mouthrinses, *S. mutans* were grown with the addition of 1% sucrose to form a mature, structured biofilm or in the absence of sucrose as a zero-biofilm control. Following biofilm formation and growth, mouthrinses were added to the biofilm wells for 15 min while being shaken orbitally to simulate swishing of the mouthrinse.

**Figure 3:**
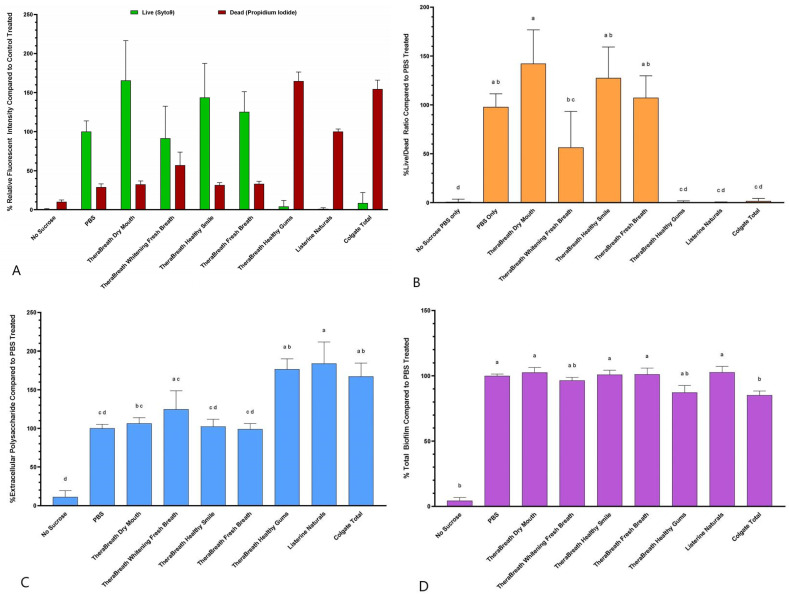
Fluorescent vitality staining and total biofilm analysis of pre-formed *S. mutans* biofilms after 15 min treatments using TheraBreath™ mouthrinses. Biofilms of *S. mutans* were grown for 18 h in the presence of Cascade Blue fluorescently labeled Dextran and subsequently treated for 15 min with mouthrinses. Following treatment, biofilm vitality was assessed using SYTO9/propidium iodide Live/Dead stain. (**A**) Live/Dead fluorescence after treatment is displayed as percentage of control treatments for each experiment. The green bar represents the percentage of the relative fluorescence of PBS and represents live cells stained with SYTO9. The red bar represents the percentage of the relative fluorescence of Listerine-treated biofilms and represents the dead cells stained with propidium iodide. (**B**) Live/Dead ratio is shown to represent the ratio of live cells to dead cells in the biofilm after treatment with mouthrinse compared to control PBS. (**C**) Extracellular polysaccharide (EPS) content of *S. mutans* biofilms after treatment was assessed by measuring incorporated fluorescently labeled dextran. (**D**) Overall biofilm remaining after treatment was measured using crystal violet staining. Statistical analysis of Live/Dead ratio, EPS, and crystal violet staining was conducted using Kruskal–Wallis test with Dunn’s multiple comparisons test comparing all mean ranks. Lowercase letters denote statistically similar treatments.

Average live and dead fluorescent intensities after treatment, normalized to treatment controls, are shown for each treatment (Figure 3A). Statistical analysis was not performed for the individual live and dead fluorescence values. Instead, Live/Dead ratios were calculated for each treated biofilm, displayed as a percentage of PBS-treated biofilms, and statistically analyzed (Figure 3B). The Live/Dead fluorescence ratio after treatment with TheraBreath™ Healthy Gums was statistically comparable to treatment with other commercially sold mouthrinses including Listerine^®^ Naturals and Colgate^®^ Total (*p* > 0.9999). This was also statistically similar to wells of *S. mutans* that were grown in the absence of sucrose. TheraBreath™ Whitening Fresh Breath also showed a statistically similar Live/Dead ratio to these mouthrinses but was not statistically different compared to PBS and other non-bactericidal TheraBreath™ mouthrinses (Dry Mouth, Healthy Smile, Fresh Breath).

To assess whether any mouthrinse affected biofilm matrix, extracellular polysaccharides (EPS) remaining within *S. mutans* biofilms after treatments were analyzed using fluorescence (Figure 3C). Of all mouthrinses tested, there was no significant disruption of biofilm structure compared to PBS treatment when assessed by fluorescently labeled dextran.

After fluorescent analysis, biofilms were stained using crystal violet to assess overall biofilm size after treatment compared to PBS-treated biofilms (Figure 3D). There were no statistically significant differences in total biofilm among the TheraBreath™ formulations or when compared to the PBS control treatment. Colgate^®^ Total had a significant reduction in total biofilm compared to PBS (*p* = 0.0496), but this reduction was not significant compared to TheraBreath™ Healthy Gums or Whitening Fresh Breath.

## 4. Discussion

The objective of this study was to determine the bactericidal and antibiofilm activity of TheraBreath™ oral rinses against *S. mutans* and saliva-derived microbial communities. Bactericidal activity was assessed by exposing planktonic and biofilm-associated bacteria to various oral rinses. Even though microorganisms, like *S. mutans*, cause issues like caries from within a protective biofilm, it is important to study antibacterial activity against planktonic *S. mutans* to establish which formulations have any measurable level of antibacterial activity because bacteria in liquid, like saliva, can potentially colonize tooth surfaces and form biofilms that are significantly more tolerant to treatments than planktonic cells [17].

The results from this study indicate that, of the TheraBreath™ oral rinses tested, only Whitening Fresh Breath and Healthy Gums demonstrated any level of antibacterial activity against *S. mutans*. Healthy Gums showed the greatest level of antibacterial activity compared to other TheraBreath™ mouthrinses based on the results for 30 and 2 min treatments of *S. mutans* in liquid. After 30 min treatments of liquid *S. mutans*, colony counts revealed that Healthy Gums killed at levels comparable to Listerine^®^ Naturals, and Whitening Fresh Breath killed at statistically comparable levels; however, Whitening Fresh Breath did not always kill 100% of input bacteria like Healthy Gums and Listerine^®^ Naturals. Healthy Gums was the only TheraBreath™ formulation to exhibit complete killing in 2 min of exposure. Measured antibacterial activity against the cariogenic bacterium *S. mutans* is an important quality for an antiseptic mouthrinse due to its association with the initiation of caries. Reduced levels of *S. mutans* in the oral cavity, especially in primary caretakers, has been shown to lower the risk of early childhood caries in their children [18,19]. Based on these results, we can argue for the use of Healthy Gums as an antiseptic mouthrinse for the prevention/control of caries in individuals at high risk of *S. mutans*-mediated dental disease.

While *S. mutans* is a pathogen that is associated with caries, it is not the only pathogen associated with the disease, nor is caries the only dental disease caused by microbes. The oral cavity consists of over 700 bacterial species of bacteria, comprising commensal, opportunistic, and pathogenic microorganisms, and thus, antibacterial activity of mouthrinses against a broad range of microbes should also be assessed [20]. Therefore, we expanded our study to consider other microorganisms that could impact the clinical efficacy of the various oral mouthrinses by testing them against salivary samples. After 30 s treatments of saliva samples, Healthy Gums exhibited comparable antibacterial activity to Listerine^®^ Naturals against saliva-derived microbes that grow aerobically on blood agar and Mitis Salivarius agar (Figure 2). While the microbiome of saliva may differ based on multiple factors, a core microbial community comprising 68 operational taxonomic units has been described in human saliva of healthy individuals [21]. Therefore, it can be inferred that saliva samples from multiple donors represent a defined range of diverse members of the saliva microbiome, many of which are aerobic or facultative, and that TheraBreath™ Healthy Gums has a broader range of antibacterial activity, beyond *S. mutans* and other oral streptococci.

It is assumed that biofilms are also significantly more tolerant of chemical disinfection than stationary-phase planktonic cells [17]. So, if a mouthrinse has antibacterial activity against planktonic cells, it cannot be assumed that it retains this activity towards biofilm-associated cells. Since acid production that affects tooth enamel comes from bacteria within a biofilm, we tested the TheraBreath™ oral rinses for antibiofilm activity against in vitro *S. mutans* biofilms. The Live/Dead fluorescence ratio after treatment with TheraBreath™ Healthy Gums was statistically comparable to treatment with other commercially sold mouthrinses including Listerine^®^ Naturals and Colgate^®^ Total (Figure 3B) indicating its ability to kill bacteria protected within a biofilm. TheraBreath™ Whitening Fresh Breath showed a statistically significant decrease in biofilm vitality compared to PBS and other TheraBreath™ rinses; however, the Live/Dead ratio was significantly higher than Healthy Gums, Listerine^®^ Naturals, and Colgate^®^ Total. Of all the mouthrinses tested, there was no significant disruption of biofilm structure when assessed by fluorescently labeled dextran and crystal violet staining (Figure 3C,D). The inability of the tested mouthrinses to reduce EPS and overall biofilm in this study reinforces the use of mouthrinses as an adjunct to physical biofilm removal, not as a replacement [8].

Overall, Healthy Gums was the only TheraBreath™ mouthrinse tested that had similar antiseptic activity to the levels of other antiseptic-claiming commercial rinses, while Whitening Fresh Breath showed intermediate antiseptic activity in some experiments. The antibacterial activity of Healthy Gums is most likely due to the active ingredient, cetylpyridinium chloride (CPC). Products that contain CPC, like Colgate^®^ Total, have shown antibacterial activity in many studies against *S. mutans* and other bacterial species [5,22,23]. CPC, a quaternary ammonium compound, disrupts bacterial cell membranes by binding to negatively charged bacterial surfaces, leading to increased membrane permeability, leakage of intracellular contents, and ultimately cell death [22]. In the context of biofilms, CPC is able to penetrate the extracellular polymeric matrix and reach bacteria located deeper within the biofilm structure [22]. This characteristic, along with its described mechanism of antibacterial activity, is a plausible reason for the antibacterial effects against *S. mutans* biofilms measured after exposure to TheraBreath™ Healthy Gums.

TheraBreath™ Whitening Fresh Breath generally exhibited less antibacterial activity compared to Healthy Gums but still showed intermediate levels. Unlike Healthy Gums, Whitening Fresh Breath lacks an obvious antimicrobial ingredient. To identify candidate ingredients that could account for the antibacterial activity of Whitening Fresh Breath, its ingredients were compared with other TheraBreath™ formulations that lacked antibacterial activity. After exclusion of similar ingredients, potential ingredients in Whitening Fresh Breath that may account for the intermediate antibacterial activity arose including sodium benzoate, polyvinylpolypyrrolidone, papain, glucose oxidase, and D-limonene.

Sodium benzoate is a preservative and has demonstrated antibacterial activity [24]; however, it is also present in other TheraBreath™ mouthrinses (Dry Mouth, Healthy Smile, and Fresh Breath), which did not show any antibacterial activity (Figure 1). Polyvinylpolypyrrolidone is a water-soluble synthetic homopolymer that is added to toothpastes and rinses to increase clearing time of hydrogen peroxide for longer whitening effects [25].

Papain, a proteolytic enzyme from papaya, is commonly used to break down proteins. The pellicle layer, being proteinaceous, also has the ability to absorb pigments, leading to stains on teeth. While it can break down pellicle proteins, this component likely did not play a role in the breakdown of extracellular matrix in our biofilm model, since *S. mutans* biofilm matrices are primarily polysaccharide [26]. The ability to reduce pellicle accumulation is not considered to be an antiseptic function, but it is a capability that aids in the overall effectiveness of mouthwash [27].

Glucose oxidase is an enzyme that converts glucose into gluconic acid and hydrogen peroxide, both of which could contribute to bacterial killing [28]. This enzyme is likely to be included in the formulation to provide a source of hydrogen peroxide since the mouthrinse does not include hydrogen peroxide. While production of hydrogen peroxide may contribute to the antibacterial activity, this enzyme requires glucose as a substrate for the production, which is present in our liquid killing assays.

Limonene, a monoterpene found in citrus oils and other plants, has been shown to have antibacterial activity, particularly in its R/D-form [29]. Also, in the presence of glucose oxidase, D-limonene is converted into the oxygenated monoterpene, carvone, which has additional antimicrobial properties [30]. While this was not tested in this study, based on these other studies, it is likely the D-limonene component of the Whitening Fresh Breath formulation is likely providing the antiseptic activity in this oral rinse.

### Limitations

While saliva-based experiments were included to better simulate clinical conditions, many of the assays were still studied in controlled in vitro settings that do not fully simulate the oral cavity or the patient. For example, some patients swish mouthrinse around very quickly, but some patients use it very slowly. Additionally, the health of the oral cavity, including saliva production and immune status, can influence how well a mouthrinse performs. Saliva composition itself can also vary between individuals due to hydration, medications, or underlying conditions, which can also impact the mouth rinse effectiveness. An additional limitation is that this study focused primarily on a single strain of *Streptococcus mutans*, but as stated, dental biofilms are composed of a complex, multispecies microbial community and the exposure time of biofilms in the experiments is longer than the directed usage times. While not an exact replica of oral biofilm, monospecies *S. mutans* biofilms are a commonly used model of oral biofilms that provides a consistent measure for initial testing of antibiofilm activity. Further research should focus on cytotoxic evaluation and the in vivo effect of TheraBreath™ mouthrinses on clinical parameters during routine usage.

## 5. Conclusions

Overall, there is limited research on the efficacy of TheraBreath™ products. When comparing the findings in this study to other commercial brands, similar trends are observed, particularly with the CPC-containing mouthrinse, TheraBreath™ Healthy Gums. While many of the tested TheraBreath™ products failed to eliminate *S. mutans* in our study, antibacterial activity may not be the main goal for the use of these products, as the specific names of the products may suggest.

## Figures and Tables

**Table 1:** Ingredients of mouthrinses used in this study.

Mouthrinse	Ingredients
TheraBreath™ Dry Mouth	Water, Glycerin, Propylene Glycol, Poloxamer 407, Xylitol, Sodium Benzoate, Flavor, Lysozyme, Amylase, Papain, Amyloglucosidase, Serralysin, Lactoferrin, Maltodextrin, *Spilanthes acmella* Flower Extract, Sodium Citrate (TheraBreath, Ewing, NJ, USA)
TheraBreath™ Whitening Fresh Breath	Water, Glycerin, Polysorbate 20, Sodium Benzoate, Polyvinylpolypyrrolidone, Natural Mint Flavor, Papain, *D*-limonene, Menthol, Glucose Oxidase (TheraBreath, Ewing, NJ, USA)
TheraBreath™ Fresh Breath	Water, PEG-40 Hydrogenated Castor Oil, Sodium Chlorite, Tetrasodium EDTA, Sodium Benzoate, Sodium Bicarbonate, Flavor, Sodium Hydroxide (TheraBreath, Ewing, NJ, USA)
TheraBreath™ Healthy Smile	Sodium Fluoride 0.05% (0.02% *w*/*v* fluoride ion) Water, Glycerin, PEG-40 Hydrogenated Castor Oil, Citric Acid, Sodium Hydroxide, Sodium Chlorite, Menthol, Citrus Lemon Peel Oil, Mentha Piperita Oil, Sodium Benzoate, Sucralose, Xylitol, Sodium Bicarbonate (TheraBreath, Ewing, NJ, USA)
TheraBreath™ Healthy Gums	Active Ingredients: Cetylpyridinium Chloride 0.05% Inactive Ingredients: Water, Glycerin, Poloxamer 407, Flavor, Sucralose (TheraBreath, Ewing, NJ, USA)
Listerine^®^ Naturals	Active Ingredients: Eucalyptol 0.092%, Menthol 0.042%, Methyl Salicylate 0.060%, Thymol 0.064% Inactive Ingredients: Water, Sorbitol, Alcohol (21.6% *v*/*v*), Poloxamer 407, Flavor, Benzoic Acid, *Stevia rebaudiana* Leaf Extract, Sodium Benzoate (Johnson & Johnson, Skillman, NJ, USA)
Colgate^®^ Total	Active Ingredients: Cetylpyridinium Chloride 0.075% Inactive Ingredients: Water, Glycerin, Propylene Glycol, Sorbitol, Poloxamer 407, Zinc Lactate, Flavor, Potassium Sorbate, Lactic Acid, Sodium Saccharin, Sucralose, Green 3, Yellow 6 (Colgate-Palmolive Co., New York, NY, USA)

## Data Availability

The original contributions presented in this study are included in the article. Further inquiries can be directed to the corresponding author.

## Appendix A

First, 24 h *S. mutans* biofilms were treated with PBS or Listerine for 4 h. Following treatment, biofilms were subjected to vitality staining using SYTO9/propidium iodide Live/Dead stain or removed by scraping, serially diluted, and plated on BHI for enumeration by colony counting. Figure A1 shows the results of colony count after treatment. Each circle represents an individual experiment. Circles crossing the *x*-axis represent biofilms with zero recovered bacteria after treatment.

Figure A2 shows live and dead relative fluorescent units (RFU) after treatment. Based on recovery of zero bacteria from Listerine-treated biofilms, live fluorescence measured in these wells was considered background staining. Figure A3 shows adjusted live fluorescence after subtraction of RFU measured in Listerine-treated biofilms from PBS-treated biofilms and Listerine-treated wells.
